# Subthalamic Nucleus Stimulation–Induced Local Field Potential Changes in Dystonia

**DOI:** 10.1002/mds.29302

**Published:** 2022-12-23

**Authors:** Christoph Wiest, Francesca Morgante, Flavie Torrecillos, Alek Pogosyan, Shenghong He, Fahd Baig, Ilaria Bertaina, Michael G. Hart, Mark J. Edwards, Erlick A. Pereira, Huiling Tan

**Affiliations:** ^1^ Medical Research Council Brain Network Dynamics Unit, Nuffield Department of Clinical Neurosciences John Radcliffe Hospital, University of Oxford Oxford United Kingdom; ^2^ Neurosciences Research Centre Molecular and Clinical Sciences Institute, St. George's, University of London London United Kingdom; ^3^ Institute of Psychiatry, Psychology and Neurosciences King's College London London United Kingdom

**Keywords:** dystonia, deep brain stimulation, subthalamic nucleus, evoked resonant neural activity, local field potentials, finely tuned gamma oscillations

## Abstract

**Background:**

Subthalamic nucleus (STN) stimulation is an effective treatment for Parkinson's disease and induced local field potential (LFP) changes that have been linked with clinical improvement. STN stimulation has also been used in dystonia although the internal globus pallidus is the standard target where theta power has been suggested as a physiomarker for adaptive stimulation.

**Objective:**

We aimed to explore if enhanced theta power was also present in STN and if stimulation‐induced spectral changes that were previously reported for Parkinson's disease would occur in dystonia.

**Methods:**

We recorded LFPs from 7 patients (12 hemispheres) with isolated craniocervical dystonia whose electrodes were placed such that inferior, middle, and superior contacts covered STN, zona incerta, and thalamus.

**Results:**

We did not observe prominent theta power in STN at rest. STN stimulation induced similar spectral changes in dystonia as in Parkinson's disease, such as broadband power suppression, evoked resonant neural activity (ERNA), finely‐tuned gamma oscillations, and an increase in aperiodic exponents in STN‐LFPs. Both power suppression and ERNA localize to STN. Based on this, single‐pulse STN stimulation elicits evoked neural activities with largest amplitudes in STN, which are relayed to the zona incerta and thalamus with changing characteristics as the distance from STN increases.

**Conclusions:**

Our results show that STN stimulation–induced spectral changes are a nondisease‐specific response to high‐frequency stimulation, which can serve as placement markers for STN. This broadens the scope of STN stimulation and makes it an option for other disorders with excessive oscillatory peaks in STN. © 2022 The Authors. *Movement Disorders* published by Wiley Periodicals LLC on behalf of International Parkinson and Movement Disorder Society.

## Graphical abstract

We found that high‐frequency stimulation‐induced spectral changes in the subthalamic local field potential such as evoked resonant neural activity, finely‐tuned gamma oscillations, broadband power suppression and 1/F exponent changes are not specific to Parkinson's disease but can also occur in isolated dystonia.

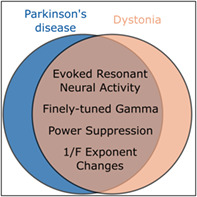

## 1 Introduction

Dystonia is a movement disorder characterised by patterned torsional movements, abnormal postures, and tremor.^1^ The prominent role of the basal ganglia‐sensorimotor network in the generation of dystonic symptoms^2^ has led to the use of deep brain stimulation (DBS) of the internal globus pallidus (GPi) in the treatment of severe and disabling generalized and segmental dystonia.^3^ Theta oscillations in GPi have been associated with dystonic symptoms and suggested as a potential signal for adaptive stimulation in dystonia.^4^, ^5^ Whereas GPi remains the DBS target of choice, subthalamic nucleus (STN) stimulation has been reported to be effective in case series of people with focal or generalized dystonia^6^ and cervical dystonia (CD).^7^, ^8^, ^9^ The rationale for using STN‐DBS in dystonia is from evidence that, besides its well‐known effectiveness on bradykinesia, rigidity, and tremor, this target is very effective against painful OFF‐period dystonia in people with Parkinson's disease (PD).^10^ Nevertheless, it is unclear if enhanced theta is also present in the STN of people with isolated dystonia.

STN stimulation in PD is associated with several spectral changes in the STN local field potential (LFP), including beta power suppression,^11^ evoked resonant neural activity (ERNA),^12^, ^13^ and finely‐tuned gamma (FTG) oscillations.^14^, ^15^ These spectral fingerprints have been linked with improvement in parkinsonian symptoms with DBS.^16^, ^17^, ^18^, ^19^, ^20^ It is unknown if these same spectral features are present during STN stimulation of people with isolated dystonia and if so how these relate to lead placement and/or clinical symptoms. Filling this knowledge gap would help understand the pathophysiology of isolated dystonia, as well as the mechanism of high‐frequency STN stimulation.

Here, we had the unique opportunity to record subthalamic LFPs from 7 patients, with idiopathic isolated CD implanted with octopolar DBS leads spanning from STN to the ventrolateral thalamus. We asked two main questions. First, how are the power spectra recorded from STN in our patients different from those reported from GPi recordings in isolated dystonia and from STN recordings in PD patients? Second, are the stimulation‐induced phenomena observed in PD patients also seen in people with dystonia, and how do these relate to lead placement?

## 2 Patients and Methods

### 2.1 Consent, Regulatory Approval, Patient Selection, and Clinical Details

This protocol was approved by the Health Research Authority UK and the National Research Ethics Service Local Research Ethics Committee (IRAS: 46576). Seven patients with isolated idiopathic dystonia were recruited at St. George's University Hospitals NHS Foundation Trust, London, and received STN‐thalamic dual targeted DBS. Written informed consent was obtained in line with the Declaration of the Principles of Helsinki. Five patients were recorded bilaterally, resulting in 12 hemispheres included in the study. Clinical details and the tested hemispheres are summarized in Table 1.

**TABLE 1:** Clinical and recording details

Patient number	Gender (m/f)	Age (y)	Diagnosis	Disease duration (y)	TWSTRS/JRS before DBS	TWSTRS/JRS at recording	Predominant symptoms	Time of recording (days post‐OP)	Data in Figures 2, 3, 4 (L/R)	FTG present (L/R)	Single pulse paradigm (L/R)
1	f	64	Segmental dystonia: CD, UL	28	18	4	Tonic posturing of the head	4	L + R	No	No
2	f	49	CD	6	26	16	Tonic posturing of the head	4	L + R	L + R	No
3	f	68	Craniocervical dystonia	55	3/8	0/1	Blepharospasm	4	R	No	No
4	f	59	CD	5	23	23	Tonic posturing of the head	5	L + R	No	No
5	f	45	Segmental dystonia: CD, UL	25	22	12	Tonic posturing of the head	7	No	L	L
6	m	52	CD	8	24	21	Tonic posturing of the head	7	L + R	No	L + R
7	m	51	Segmental dystonia: CD, trunk	10	26	18	Tonic posturing of the head	7	L + R	No	L + R

Abbreviations: TWSTRS, Toronto Western Spasmodic Torticollis Rating Scale (range: 0–35); JRS, Jankovic Rating Scale; DBS, deep brain stimulation; L, left; R, right; FTG, finely tuned gamma; CD, cervical dystonia; UL, upper limb; post‐OP, post‐operation.

### 2.2 Surgery and Lead Localization Assessment

**FIG 1:**
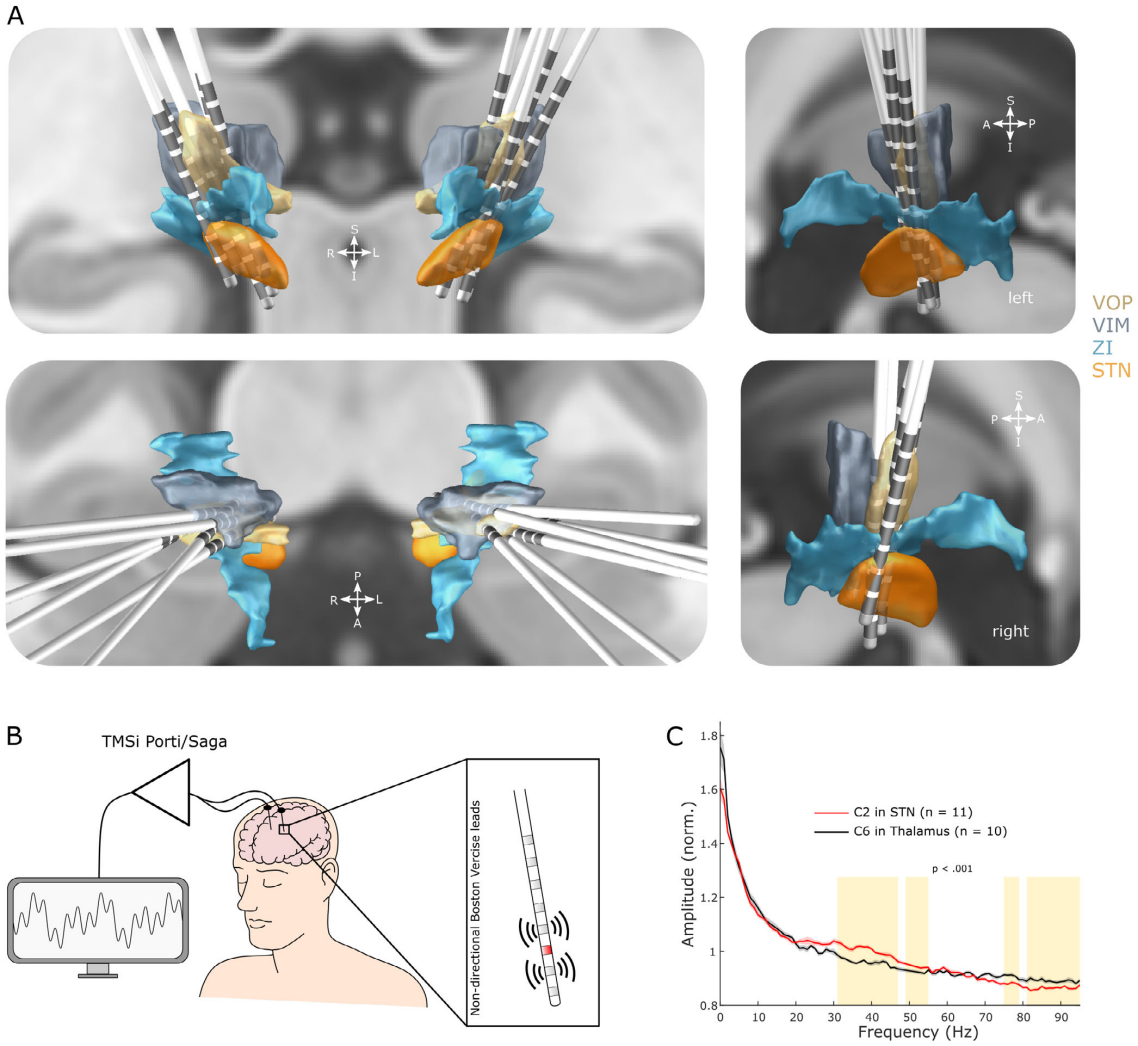
Recording setup and DBS (deep brain stimulation) dual targeting. (**A**) Lead reconstructions of all 12 electrodes used in this study. STN, subthalamic nucleus; ZI, zona incerta; VIM, nucleus ventralis intermedius of the thalamus; VOP, nucleus ventralis oralis posterior of the thalamus. (**B**) Leads were temporarily externalized, and local field potentials (LFPs) were recorded 4 to 7 days after implantation using a CE‐marked amplifier system. The six middle contact levels were successively stimulated (red), which allowed bipolar LFP recordings from the two adjacent contact levels. (**C**) Power spectral densities (PSD, mean ± standard error of the mean) from the C2 contact in STN (as determined by Lead‐DBS, *n* = 11 hemispheres) and the C6 contact in ventrolateral thalamus (*n* = 10) without stimulation. Clusters that are significantly different (cluster‐based permutation test, *P* < 0.001) between STN and thalamus are highlighted in amber. (CE, conformity with European health, saftey and environmental protection standards) [Color figure can be viewed at wileyonlinelibrary.com]

The surgical targets were the ventrolateral thalamus (nucleus ventralis intermedius [VIM] and nucleus ventralis oralis posterior [VOP]), rostral Zona incerta (rZI), and STN.^21^ The nondirectional Vercise Standard Lead (model 2201, Boston Scientific Corporation, Marlborough, MA, USA) with eight‐ring contacts (length, 30 cm; diameter, 1.3 mm; contact spacing, 0.5 mm; contact length, 1.5 mm; and contact span, 15.5 mm) was implanted such that inferior contacts were placed in STN, superior contacts in ventrolateral thalamus, and intervening contacts within or close to the rZI. Electrodes were implanted and externalized, and lead trajectories were reconstructed (Fig. 1A) as described before.^13^

### 2.3 Stimulation and Data Recording

Data were recorded between four and seven days postoperatively (Table 1), when electrode leads were externalized and patients were *off* all anti‐dystonic medication. Monopolar high‐frequency stimulation was tested at the six middle contacts (C2–C7), as described before.^13^ LFPs and EMGs from the affected neck muscles were amplified and sampled at 4096 Hz using a TMSi Saga (TMSi International, Oldenzaal, Netherlands), and custom‐written software was developed using the C programming language (Fig. 1B).

### 2.4 Experimental Paradigm

In patients 1–4, 6, and 7 (11 hemispheres), each of the six middle contacts was continuously stimulated at 130 Hz with an increase in intensity from 0.5 to 4.5 mA or until side effect threshold was reached in steps of 0.5 mA (see Fig. 3A). Each DBS block lasted for 46.92 ± 0.99 seconds (mean ± standard error of the mean) separated by resting periods of 27.50 ± 0.59 seconds. In addition, different stimulation frequencies (100, 130, 150, and 180 Hz for 2 minutes each) were tested in patient 2 using the contact and current that elicited the most prominent ERNA without side effects. In patients 5 to 7, single‐pulse stimulation was subsequently applied at 2 mA (patient 5) or 4 mA (patients 6 and 7) to all eight contact levels (25 pulses to all contacts), and the remaining seven contacts were recorded in unipolar mode. If not indicated differently, a stimulation frequency of 130 Hz was used.

### 2.5 Signal Processing

#### 2.5.1 Preprocessing and Time Frequency Decomposition

All data were analyzed using custom‐written scripts in MATLAB (version 2020b, The MathWorks Inc., Natick, MA, USA). Continuous LFP signals were high‐pass filtered at 1 Hz and notch‐filtered at 50 Hz (second‐order IIR notch filter). Spectral amplitudes were estimated between 1 and 500 Hz using the short‐time fast Fourier transform with a window length of 1 second, 25% overlap of consecutive windows, and a Hamming window yielding a frequency resolution of 1 Hz.

#### 2.5.2 Power Changes and Aperiodic Exponents

**FIG 2:**
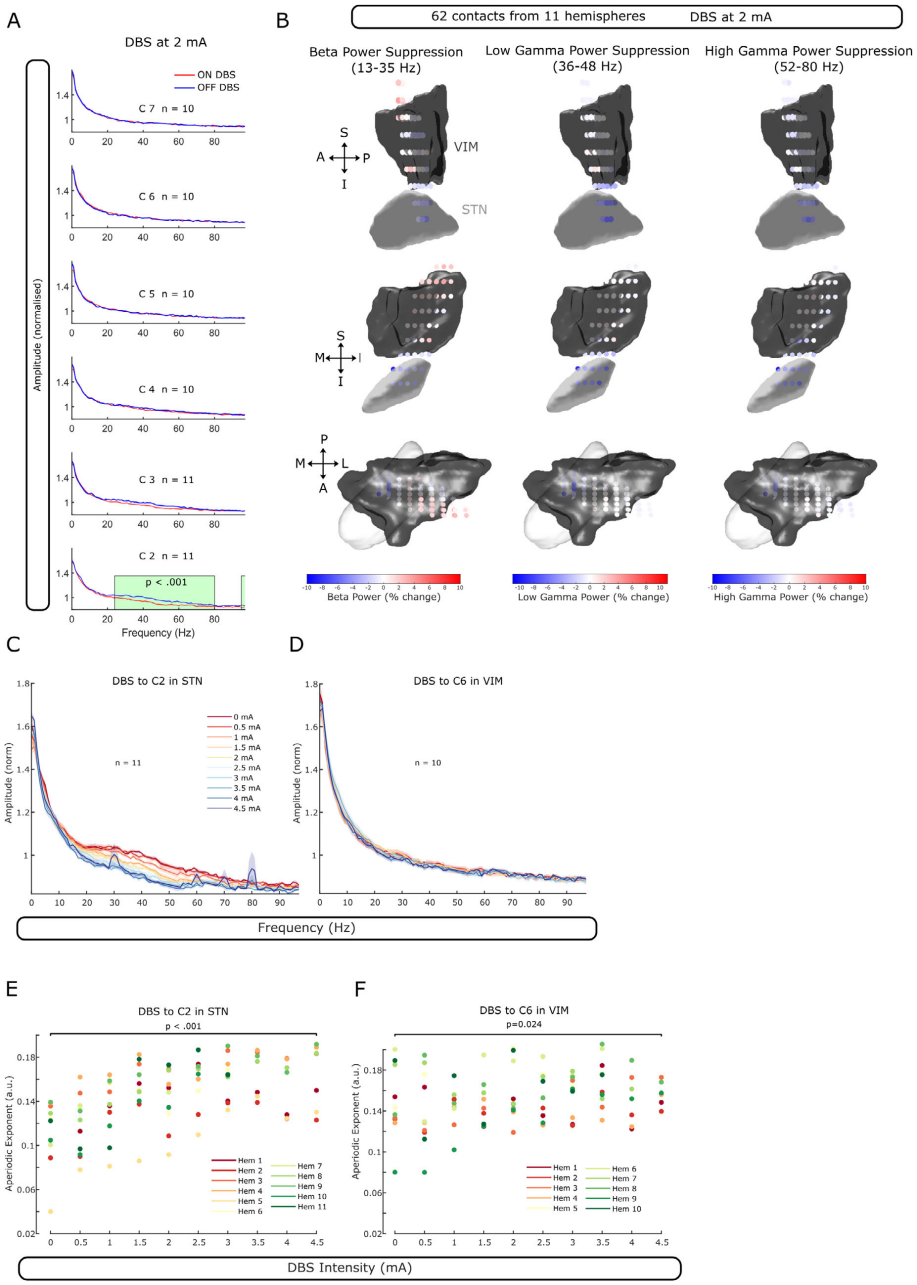
DBS (deep brain stimulation)‐induced power suppression in STN‐LFPs (subthalamic nucleus‐local field potential) of dystonic patients. (**A**) When stimulating contacts C2 to C7 at 2 mA and recording from the adjacent contact pair, average power (mean ± standard error of the mean) in the beta (24–35 Hz) and gamma (36–80 Hz) range was suppressed compared to baseline in the most inferior contact only (in STN). Significant clusters are highlighted in green (cluster‐based permutation test, *P* < 0.001). (**B**) Beta (13–35 Hz), low‐gamma (36–48 Hz), and high‐gamma (51–80 Hz) power suppression is strongest in the dorsolateral part of STN (*n* = 62 contacts). (**C**) Average power between ~20 and ~80 Hz is increasingly suppressed in STN with increasing DBS intensity. Note the artifacts of stimulation at 4.5 mA manifesting as prominent peaks in different frequency bands (at ~30, 60, 70, and 80 Hz). (**D**) No power suppression with increasing DBS intensity when VIM is stimulated. (**E**) Aperiodic exponents of the power spectrum increase (1/F slope on a log–log scale becomes steeper) in STN‐LFPs with increasing DBS intensity (LME: estimate = 0.006, *t* = 8.15, *P* < 0.001). (**F**) Aperiodic exponents of VIM‐LFPs increase only slightly with increasing DBS intensity (LME: estimate = 0.002, *t* = 2.30, *P* = 0.024) (Hem: hemisphere). [Color figure can be viewed at wileyonlinelibrary.com]

Power spectral densities (PSDs) from 1 to 95 Hz were calculated for LFPs recorded in bipolar montage and each DBS setting (Fig. 2). We defined a 30‐second epoch before the first DBS block as baseline. Power of ~50 Hz artifact of mains interference was removed (48–52 Hz), and the gap in the PSD was linearly interpolated. PSDs of both the baseline and all DBS blocks were normalized to the mean power between 1 and 95 Hz of the baseline PSD. Percentage change in the power relative to the baseline for three frequency ranges was calculated: beta (13–35 Hz), low gamma (36–48 Hz), and high gamma (52–80 Hz).

Aperiodic exponents were isolated from the PSD using the open‐source FoooF algorithm (version 1.0.0).^22^ Settings for the algorithm were set as follows: peak width limits: 2–12; maximum number of peaks: *infinite*; minimum peak height: 0; peak threshold: 2; and aperiodic mode: *fixed*. Power spectra were parameterized across the frequency range of 5 to 50 Hz. The lower bound was selected to avoid the impact of low‐frequency oscillations, and the upper bound was selected to avoid the impact of spectral plateaus at high DBS intensity.^23^ The same FoooF settings were used to identify meaningful changes in the aperiodic exponent of STN‐LFPs with dopaminergic medication and high‐frequency stimulation.^24^

#### 2.5.3 ERNA Analysis

**FIG 3:**
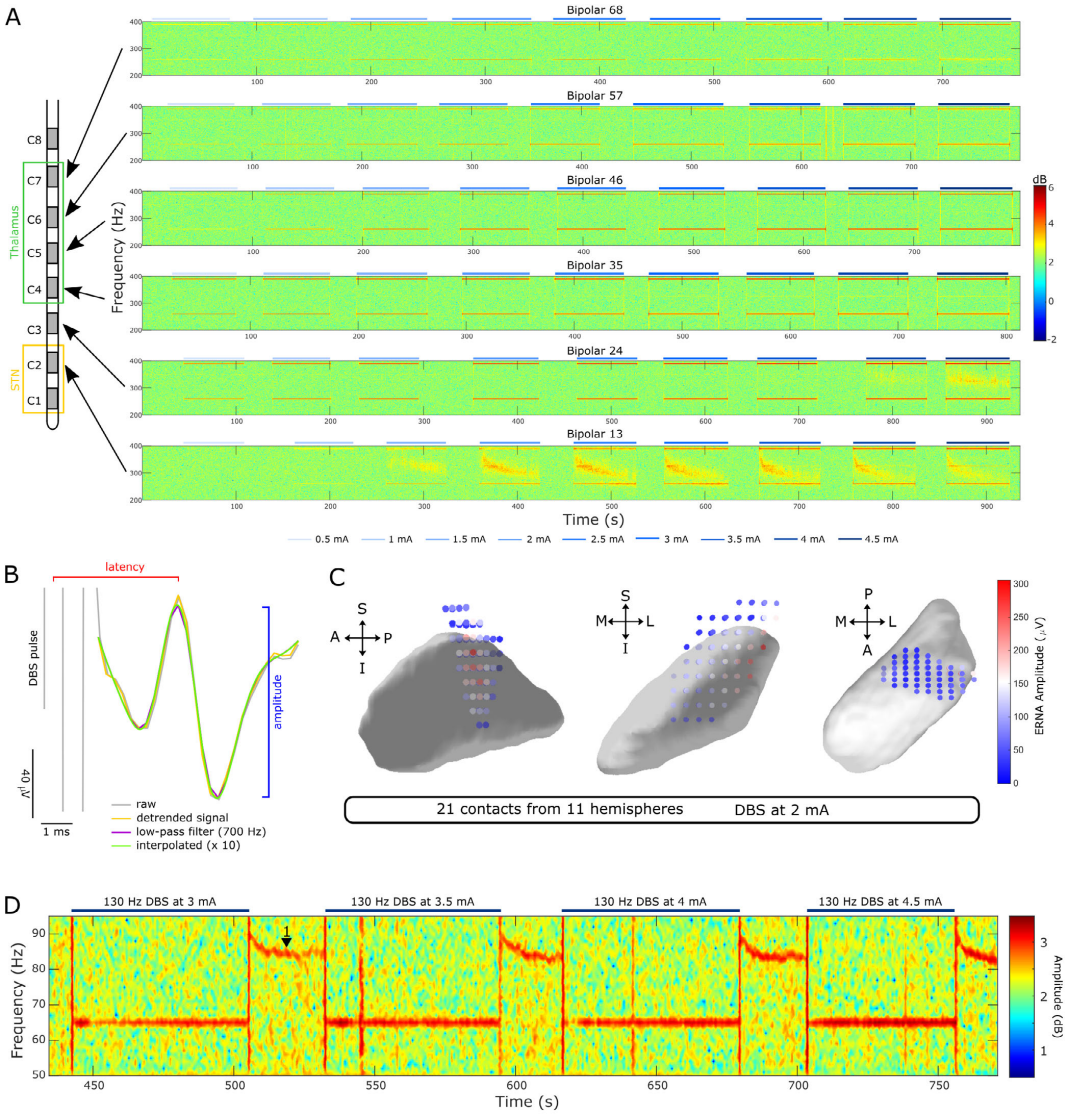
Evoked resonant neural activity (ERNA) and FTG (finely‐tuned gamma) can be elicited by STN‐DBS (subthalamic nucleus‐deep brain stimulation) in dystonia. (**A**) Stimulating contacts within or close to STN (C2) elicits ERNA recorded from adjacent contacts (C1–C3) with increasing DBS intensity (example spectrograms shown for patient 1). Note that higher DBS intensity is required to elicit ERNA in contact levels that are farther from a sweet spot in STN (DBS to C3 and recording from C2 to C4). Stimulation to any of the more superior contacts (in thalamus) did not elicit ERNA (C4–C7). (**B**) Schematic of how ERNA latency and amplitude were calculated based on the time series waveform. The inter‐pulse interval was linearly detrended, low‐pass filtered at 700 Hz, and upsampled using a spline interpolation. (**C**) Average ERNA amplitudes of the first 10 pulses at 2‐mA stimulation are largest within dorsolateral STN (*n* = 21; contacts from the right hemisphere were mirrored to the left, individual contact positions were interpolated in 3D as in Horn et al^46^) (S: superior, I: inferior, A: anterior, P: posterior, M: medial, L: lateral). The three subplots show the left STN viewed from lateral, anterior, and superior, respectively. (**D**) Spectrogram with blocks of increasing DBS intensity (3–4.5 mA). DBS‐induced FTG (label 1) occurs after the respective DBS blocks (3 of 12 hemispheres) when patients do not exhibit prominent dystonia due to the stun effect. [Color figure can be viewed at wileyonlinelibrary.com]

The presence of the ERNA was assessed in two ways. In the power spectrum, it was defined as a high‐amplitude and high‐frequency activity that starts at ~350 Hz and decreases in frequency and amplitude with sustained stimulation^13^ (see contact C2, Fig. 3A). In the time series waveform, the ERNA was defined as a high‐amplitude evoked response between two stimulation pulses (Fig. 3B), which resonates as DBS is switched off or paused.^13^

To map ERNA amplitudes onto subcortical space, stimulation contact positions in MNI (Montreal Neurological Institute) space (MNI 152 2009b NLIN ASYM) were extracted from the Lead‐DBS pipeline. To calculate ERNA amplitudes, the inter‐pulse interval was linearly detrended, low‐pass filtered at 700 Hz, and upsampled by factor 10 using a spline interpolation (Fig. 3B). ERNA amplitudes following the first 10 pulses at 2 mA were averaged because this was the highest intensity that was tested across all hemispheres. Average ERNA amplitudes of the first 10 pulses were assigned to their respective stimulation contacts in MNI space and linearly interpolated in three‐dimensional space (Fig. 3C). Only contacts with a clear ERNA peak were included in the analysis, which resulted in 21 contacts shown in Figure 3C. Contacts from the right STN were mirrored to the left and are presented in one combined image.

#### 2.5.4 FTG

The post‐DBS FTG was identified after each stimulation block at increasing intensity (see Fig. 3D), analyzed as described before,^25^ and baseline normalized to the mean amplitude of all power estimates between 1 and 95 Hz of a 30‐second period before the first stimulation block.

#### 2.5.5 Evoked Neural Activity after Single‐Pulse Stimulation

In patients 5 to 7, we studied evoked neural activities (ENAs) after single‐pulse stimulation with an average inter‐pulse interval of 3.97 ± 0.04 seconds. To quantify ENAs, LFPs were aligned to individual pulses and averaged. We extracted ENA amplitudes and latencies after every DBS pulse, as shown in Figure 3B.

### 2.6 Statistics

Statistical analyses were conducted using custom‐written scripts in MATLAB. Linear mixed‐effect models were used to assess the effect of stimulation contacts on ENA parameters and aperiodic exponents as described before.^13^ To identify clusters of significant power suppression during DBS, we used nonparametric permutation tests with 1000 permutations. Only clusters with *P* < 0.001 are highlighted in Figures 1C and 2A.

## 3 Results

### 3.1 Subthalamic and Thalamic Power Spectral Densities in Dystonia

We recorded blocks of 37.88 ± 4.86 seconds at rest without any stimulation from 10 (thalamus) and 11 (STN) hemispheres and did not observe prominent peaks in the theta (4–8 Hz) or beta (13–35 Hz) range in the power spectra of both STN and thalamus (Fig. 1C). There was no correlation between muscle activity recorded from the dystonic muscles and LFP power of any frequency range in either STN or thalamus at rest. When comparing power spectra recorded from within or close to STN (recorded from the C2 contact) and thalamus (C6), amplitudes were larger in STN from 31 to 55 Hz and vice versa from 75 to 95 Hz (cluster‐based permutation test, *P* < 0.001, Fig. 1C).

### 3.2 DBS Induces a Broad Power Suppression and increases the aperiodic exponent in the STN LFPs of Dystonic Patients

Continuous 130‐Hz STN‐DBS (delivered to the C2 level) at 2 mA induced power suppression over a broad frequency range (25–80 Hz, *P* < 0.001, recorded from the adjacent contacts), whereas stimulation to any of the more superior contacts in rZI and thalamus did not lead to significant power changes (in adjacent contacts, Fig. 2A). Power suppression of the beta, low‐gamma, and high‐gamma range was strongest in the dorsolateral part of STN and decreased in all directions (62 contacts from 11 hemispheres included, Fig. 2B). Consistent with this, power suppression in STN was more pronounced with increasing DBS intensity (Fig. 2C), whereas increasing DBS currents in VIM/VOP thalamus did not affect the PSD (Fig. 2D).

Recently, the aperiodic exponent (1/F slope of the PSD) was suggested as a marker for E/I balance.^24^, ^26^, ^27^ We extracted the aperiodic exponent between 5 and 50 Hz to avoid a spectral plateau at ~50 Hz and found that aperiodic exponents in STN increase with increasing DBS currents (Linear mixed‐effect model (LME): estimate = 0.006, *t* = 8.15, *P* < 0.001, *n* = 11 hemispheres), consistent with the hypothesis that high‐frequency stimulation inhibits STN (Fig. 2E). This process appears to be nonlinear and to flatten out with further increasing currents. In contrast, aperiodic exponents in VIM‐LFPs were less affected by increasing intensity of VIM‐DBS (LME: estimate = 0.002, *t* = 2.30, *P* = 0.024, *n* = 10, Fig. 2F).

### 3.3 ERNA and Finely‐Tuned Gamma Can Be Elicited by STN‐DBS in Dystonia

When stimulation was delivered at 130 Hz to contacts within or close to the STN (C2), we observed ERNA recorded from neighboring contacts progressively with increasing DBS intensity in 11 of 12 tested hemispheres (Fig. 3A, lowest panel). When the next higher contact (C3) was stimulated, ERNA was still recorded from adjacent contacts, however only at much higher DBS intensity (eg, 4.5 mA for patient 1, left hemisphere). Stimulation to any of the superior contacts in thalamus did not elicit ERNA recorded from surrounding contacts. The observed ERNA shows similar characteristics as reported before in PD: it starts as a high‐frequency oscillation at ~350 Hz and gradually decreases before reaching a steady state after 1 minute.^13^

ERNA has previously been suggested as a placement marker for STN. To support this, average ERNA amplitudes of the first 10 pulses of 130‐Hz DBS (2 mA) were largest in dorsolateral STN (Fig. 3C) and decreased in all directions with increasing distance from that sweet spot (ERNA from 21 contacts and 11 hemispheres was included).

Levodopa‐induced FTG was suggested as a biomarker for dyskinesia in PD.^28^ Recently, we reported that FTG can also be induced in STN by DBS alone without dopaminergic medication or dyskinesia in PD patients.^25^ Now, we observed the same phenomenon in two dystonic patients (Fig. 3D). Unlike previous reports of FTG in dystonia,^29^, ^30^ this FTG activity is *de*‐*novo* DBS‐induced and not present at rest before stimulation. In these patients, DBS‐induced FTG appears when stimulation is switched OFF and decreases in frequency as reported before.^25^ We observe DBS‐induced FTG in 2 of 7 patients (3 of 12 hemispheres), a similar proportion compared to what was reported in PD.^25^ DBS‐induced FTG did not correlate with dystonic symptoms or the clinical effect of STN‐DBS.

### 3.4 Single‐Pulse Stimulation Induces ENA, and Its Parameters Change with Distance from the STN

**FIG 4:**
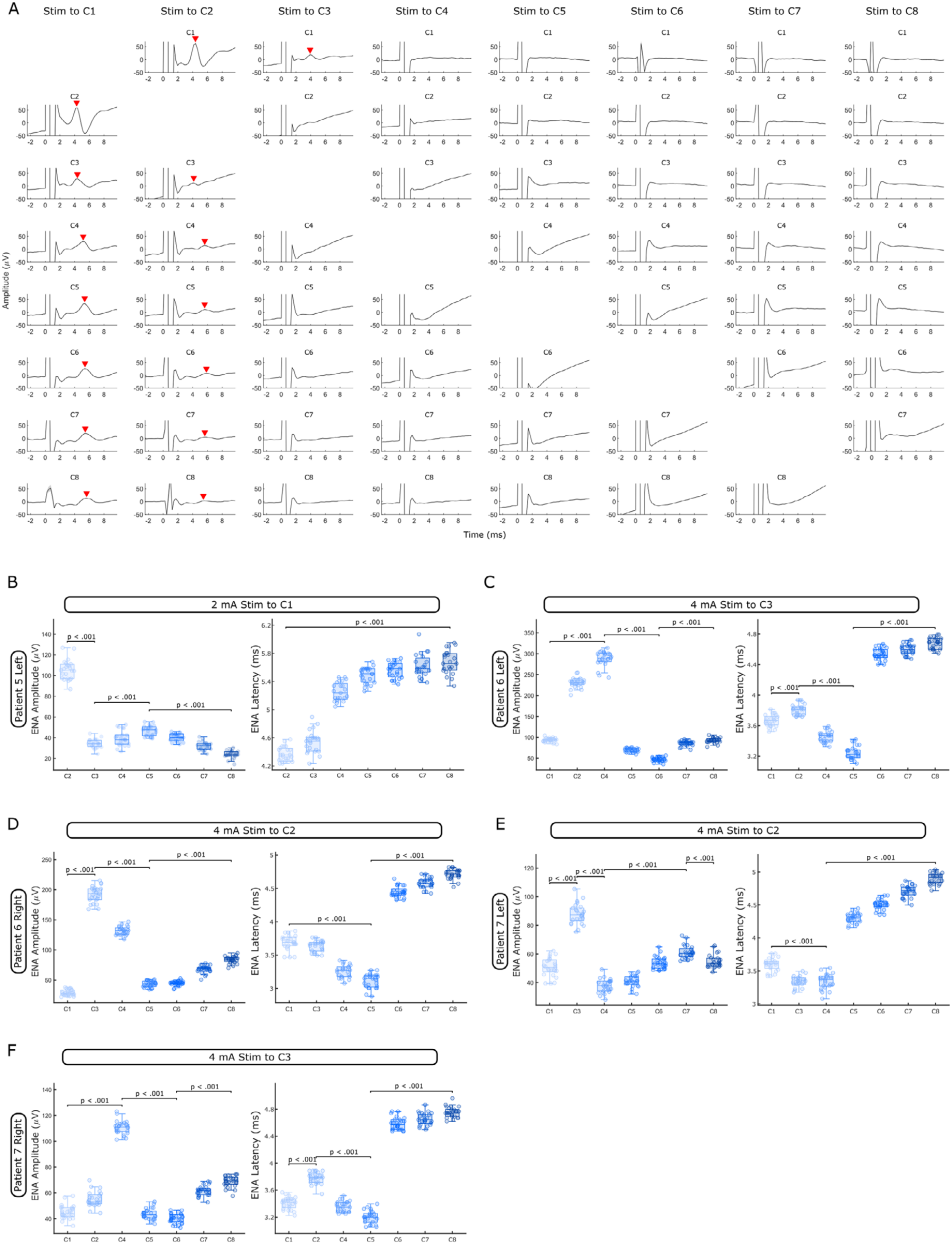
Single‐pulse STN (subthalamic nucleus) stimulation elicits evoked neural activity (ENA). (**A**) Average ENA responses (mean ± standard error of the mean; *n* = 23 when Stim to C1 and *n* = 25 for all other columns) after single DBS (deep brain stimulation) pulses at 2 mA (ENA peaks are highlighted with red arrowheads, data from patient 5 left lead). (**B–F**) When stimulating a contact in STN (C1 in B, C2 in D and E, C3 in C and F, as determined by Lead‐DBS), ENA amplitudes were largest in the neighboring contacts (in C–F: superior contact > inferior contact), decreased outside of STN, and increased again in the thalamus. ENA latencies were shortest in contacts adjacent to stimulation (B) or decreased initially with increasing distance (C–F). Latencies then increased between C3 and C4 (B), C5 and C6 (C, D, and F), and C4 and C5 (E), a jump that mirrors the transition from zona incerta to thalamus (*P*‐values of linear mixed‐effect models are shown). [Color figure can be viewed at wileyonlinelibrary.com]

To test if and to what extent STN‐DBS will affect neurons in rZI and thalamus, we applied repetitive single‐pulse stimulation in patients 5–7 (Table 1) to all eight contact levels and recorded LFPs from the remaining seven contacts in unipolar mode. When aligning and averaging the evoked neural activity (ENA) over successive DBS pulses, we observed a clear ENA peak in all contacts when stimulating the two most inferior contact levels in or close to STN (C1 and C2, Fig. 4A). However, the pattern of the ENA changed across different recording contacts. In inferior contacts within or close to STN (C1 + C2), there was an “oscillatory” pattern comprising a peak and a trough. In the remaining contacts (C3–C8), we observed only a peak whose latency increased with distance from STN (LME: estimate = 0.24, *t* = 24.92, *P* < 0.001). This process appeared to be nonlinear with the sharpest increase between C3 and C4, which mirrors the transition from rZI to thalamus (Fig. 4B). Furthermore, ENA amplitudes changed as a function of distance from STN. When the most inferior contact in STN (C1) was stimulated, ENA amplitudes (calculated as shown in Fig. 3B) were largest in C2 and decreased in C3 (LME: estimate = −71.23, *t* = −44.72, *P* < 0.001) before increasing again up to the C5 contact, which is placed in the thalamus (LME: estimate = 6.10, *t* = 9.18, *P* < 0.001) (Fig. 4B). ENA amplitudes decreased again in any of the more superior contacts (C6–C8) (LME: estimate = −7.63, *t* = −24.72, *P* < 0.001). Changing DBS pulse polarity did not reverse the polarity of the ENA after each pulse. Single‐pulse stimulation in patients 6 and 7 had similar effects. In these patients, contact C2 or C3 was placed in STN (as determined by lead reconstructions, see Fig. 4C–F). Again, ENA amplitudes were largest in contacts adjacent to stimulation (higher amplitudes in the superior neighbor), then decreased in the middle contacts (C4–C6), and increased again in contacts placed in the thalamus (C7 and C8). ENA latencies were lowest in the middle contact levels (C4 and C5) and rapidly increased between C5 and C6 (Fig. 4C, D, and F) or between C4 and C5 (Fig. 4E), a jump that mirrors the transition from rZI to thalamus.

## 4 Discussion

Here, we report for the first time stimulation‐induced spectral changes in STN activity in people with isolated dystonia. We did not observe prominent theta peaks at rest as has previously been reported in recordings from GPi. However, we found a range of robust stimulation‐induced changes in our dystonia cohort that have previously been reported only in PD, such as broadband power suppression, ERNA, and DBS‐induced FTG, which are specific to STN and not observed in the thalamus. ERNA/ENA amplitudes and DBS‐induced broadband suppression were largest in STN, confirming their usefulness as markers for lead placement in STN and contact selection in general. Finally, we showed that the aperiodic exponent of STN‐LFPs changes with increasing DBS intensity such that it is reconcilable with the E/I hypothesis. Overall, these findings point to potential differences in network dynamics in dystonia between GPi and STN, which may be relevant to future work studying correlations of spectral features with clinical symptoms for adaptive stimulation in dystonia. Furthermore, STN‐DBS‐induced spectral changes reported here may reflect a transdiagnostic pattern of how STN‐LFPs respond to high‐frequency stimulation. Understanding the origin and temporal dynamics of these changes may open a door to understanding mechanisms underlying high‐frequency STN stimulation, in general, and therefore its potential application to treatment of other neurological and psychiatric disorders.

### 4.1 Spectral Features of the Dystonic STN and Thalamus

Theta oscillations in GPi^4^, ^5^, ^31^, ^32^, ^33^ and STN^34^ have been associated with dystonic symptoms and, therefore, suggested as a potential signal for adaptive stimulation in dystonia. Here, we did not observe a prominent peak in the theta frequency band of the STN‐LFP at rest, which differs from the studies mentioned earlier in GPi and STN (Fig. 1C). Despite the postoperative stun effect as a potential confounder in this study, our results are confirmed by a previous report that did not find increased theta power in the STN of dystonia patients, which could be due to differences in input between the GPi and STN.^35^ Another recent study indicates that dystonia‐related spectral changes in the STN may be more prominent during voluntary movements; however, this study investigated dystonia as a motor sign of PD instead of isolated dystonia.^36^

Contrary to the prominent beta peaks in STN that are a hallmark of PD,^37^, ^38^ we did not observe a clear beta peak in the STN of dystonic patients (Fig. 1C), despite other studies showing beta activity in the STN of dystonic patients at rest^35^ and subthalamic beta peaks in patients with obsessive compulsive disorder.^39^ However, we observed larger beta power in STN compared to the thalamus. Two previous studies reported higher beta peaks in the GPi‐LFP of PD compared to dystonia.^33^, ^40^ These disease‐specific spectral differences may be explained by the widespread neurodegeneration in PD, which affects basal ganglia neurophysiology and cements excessive beta synchronization within the basal ganglia as a near‐pathognomonic marker of bradykinesia and rigidity in PD.

### 4.2 DBS‐Induced Power Suppression, ERNA, and DBS‐Induced FTG Are Not Specific to PD

Stimulation‐induced changes in STN‐LFPs in the beta and gamma range are well studied in PD.^13^, ^16^ What is not clear is if these changes are specific to PD or common features of how the STN responds to high‐frequency stimulation. Our results support the latter. From the broad (25–80 Hz) subthalamic DBS‐induced power suppression (Fig. 2A), we can infer that excessive, pathological synchronization in STN anywhere in this broad spectrum in a particular disease could be flattened by high‐frequency STN stimulation. Along with our finding of increasing aperiodic exponents as a function of DBS intensity, we could infer that high‐frequency STN‐DBS will overall have an inhibitory effect on STN neuronal activity. This might broaden the scope of DBS and make STN‐DBS a viable option for other disorders with excessive oscillatory peaks in the STN power spectrum.

As in PD, we also observed ERNA in the STN of dystonic patients. Several attempts have been undertaken to find clinical correlates of the ERNA in PD.^13^, ^18^, ^19^, ^41^ Although its origin is unclear, it is assumed that the ERNA originates from the effect of STN‐DBS on reciprocal STN–GPe (external globus pallidus) connections.^18^ Here, we provide evidence that the ERNA is not reliant on PD‐specific changes but can be elicited in the brains of dystonic patients without widespread neurodegeneration. However, it is still possible that the ERNA is modulated in the dopamine‐deficient brain similar to beta activity.

Levodopa‐induced FTG has previously been recorded in PD and related with the presence of dyskinesia.^28^ This relation was recently challenged by a study showing that DBS‐induced FTG can be observed in PD even without dyskinesia.^15^, ^25^ Previously, spontaneous FTG has also been reported in dystonic patients in both cortical and thalamic LFPs, when it was related with hyperkinetic movements.^29^, ^30^ In our cohort, we recorded *de*‐*novo* DBS‐induced FTG in STN, which did not vary relative to dystonic postures but appeared reliably after DBS was stopped. This challenges the direct relation between DBS‐induced FTG and both Parkinsonian and dystonic symptoms, and it may rather present a signal of effective STN stimulation,^15^ be it epiphenomenal or not.

### 4.3 ERNA/ENA Amplitudes and DBS‐Induced Power Suppression Are Largest within STN

Both ERNA^12^, ^42^ and beta power^43^, ^44^ have been suggested as placement markers during surgery and as predictors for the best clinical contact. Our findings of the largest ERNA amplitudes and strongest beta/gamma power suppression in the dorsolateral part of STN (Figs. 2B and 3C) are in line with the aforementioned studies. Importantly, we show that amplitudes and latencies of ENAs after single DBS pulses are also localized to STN (Fig. 4). It is, therefore, not necessary to apply longer bursts of stimulation. Single pulses, which can be applied in a fraction of a second, may be sufficient to optimize lead placement and accelerate contact selection. In general, ENA and ERNA represent the same activity, but ERNA has the added resonant effect caused by repetitive stimulation pulses at an inter‐pulse interval (frequency) that enhances the amplitude and duration of the ENA (positive interference). Moreover, ENA amplitudes and latencies do not decrease linearly as a function of distance on stimulation of STN (Fig. 4B). ENA amplitudes are largest in STN, second largest in contacts placed in the ventrolateral thalamus, and lower in between (see Fig. 4B,E). This possibly indicates the thalamic region that receives most inputs from the basal ganglia output structures.

Overall, our results confirm the utility of ERNA/ENA amplitudes and beta/gamma power suppression for both lead placement and contact selection but challenge the specificity of these spectral changes for PD.

### 4.4 Limitations

Our results may have been confounded by postoperative stun effect, which obscured dystonic symptoms and is known to lower beta activity in PD.^45^ Furthermore, we have a relatively low sample size of 7 dystonic patients (12 hemispheres). This is mostly due to the limited number of externalized DBS patients, and externalized dystonic patients with leads in STN are even rarer.

## Author Contribution

Conceptualization: H.T., F.T., E.P., F.M., and C.W. Data statistics and analysis: C.W. Acquisition of data: C.W., F.B., I.B., S.H., and F.T. Software: A.P. Clinical data resources: F.M., M.H., and E.P. Writing—original draft: C.W. Writing—review and editing: H.T., C.W., F.T., and M.E. Visualization: C.W. Supervision: H.T. and F.T. Funding acquisition: H.T. and E.P. All authors read and approved the final manuscript.

## Data Availability

All data will be available from the Medical Research Council Brain Network Dynamic Unit data sharing platform: https://data.mrc.ox.ac.uk/
